# MosquitoMap and the Mal-area calculator: new web tools to relate mosquito species distribution with vector borne disease

**DOI:** 10.1186/1476-072X-9-11

**Published:** 2010-02-18

**Authors:** Desmond H Foley, Richard C Wilkerson, Ian Birney, Stanley Harrison, Jamie Christensen, Leopoldo M Rueda

**Affiliations:** 1 Division of Entomology, Walter Reed Army Institute of Research, 503 Robert Grant Avenue, Silver Spring, MD 20910, USA; 2 Worldview Solutions Inc. 101 South 15th Street, Suite 104, Richmond, VA 23219. USA

## Abstract

**Background:**

Mosquitoes are important vectors of diseases but, in spite of various mosquito faunistic surveys globally, there is a need for a spatial online database of mosquito collection data and distribution summaries. Such a resource could provide entomologists with the results of previous mosquito surveys, and vector disease control workers, preventative medicine practitioners, and health planners with information relating mosquito distribution to vector-borne disease risk.

**Results:**

A web application called MosquitoMap was constructed comprising mosquito collection point data stored in an ArcGIS 9.3 Server/SQL geodatabase that includes administrative area and vector species x country lookup tables. In addition to the layer containing mosquito collection points, other map layers were made available including environmental, and vector and pathogen/disease distribution layers. An application within MosquitoMap called the Mal-area calculator (MAC) was constructed to quantify the area of overlap, for any area of interest, of vector, human, and disease distribution models. Data standards for mosquito records were developed for MosquitoMap.

**Conclusion:**

MosquitoMap is a public domain web resource that maps and compares georeferenced mosquito collection points to other spatial information, in a geographical information system setting. The MAC quantifies the Mal-area, i.e. the area where it is theoretically possible for vector-borne disease transmission to occur, thus providing a useful decision tool where other disease information is limited. The Mal-area approach emphasizes the independent but cumulative contribution to disease risk of the vector species predicted present. MosquitoMap adds value to, and makes accessible, the results of past collecting efforts, as well as providing a template for other arthropod spatial databases.

## Background

Mosquitoes are required for the natural transmission of important diseases such as malaria, dengue, Japanese encephalitis, Yellow fever, West Nile, lymphatic filariasis, and Chikungunya. Knowing when and where mosquito disease vectors occur could be vital information to combat these diseases. Over 3,500 mosquito species are currently formally recognized, but only a minority transmit disease, and these vectors vary geographically in their medical importance. A first step toward understanding mosquito distribution is to gather high quality taxonomic and geographical information about mosquito occurrence. Creating a computerized database of mosquito collection records is not a new idea [1,2], but technological advances such as the Internet make a distributed database more achievable.

Suitable mosquito collection data can be retrieved from museum specimens, from records maintained by mosquito control agencies, or from the scientific literature. Foley et al. [3] demonstrated the value of museum mosquito collection records for understanding mosquito biogeography and ecology, and for planning mosquito surveys. The basic information required are the longitude and latitude, the species identification, and date of the mosquito collection. Other information, such as on the habitat, add value to the record, and Foley et al. [4] listed over 60 fields of information about a collection event that could be recorded. These authors proposed that standards be adopted for recording collection data, because of the growth and interoperability of online inventories such as the Global Biodiversity Information Facility (GBIF) [5].

Point collection data can be matched to remotely sensed data or climatic averages to develop mosquito species-specific models of distribution or habitat suitability [6,7]. Similarly, pathogen or disease suitability models have been developed [8] that can be fine-tuned with more detailed vector information. Ecological niche modeling has been identified as a broadly applicable method for disease studies, including for predicting interactions among participating species [9]. More specifically, if the generalized spatial extent of a mosquito vector, human host and pathogen can be approximated for an area of interest (AOI), and the extent of co-occurrence (aka the Mal-area [10]) quantified, then the value for different AOI could be compared to provide a simplified estimate of relative disease risk. However, any attempt to understand disease risk by this method should account for the diversity of vector species within the particular AOI.

Here we describe the development of an online spatial database for mosquito collection records and distribution models called MosquitoMap [11] that we designed for medical entomologists, vector disease control workers, preventative medicine practitioners, and health planners to promote knowledge about mosquito distribution. We also describe a unique tool within MosquitoMap, called the Mal-area calculator (MAC) that relates the distribution of vectors, humans and pathogens/disease.

## Methods

### MosquitoMap website

**Figure 1:**
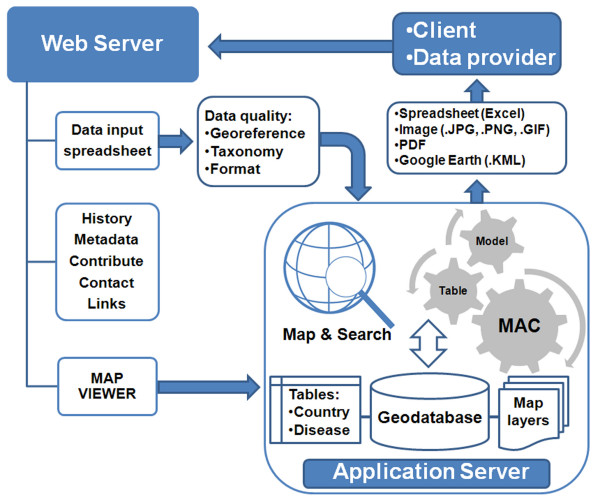
**System architecture of MosquitoMap**. A client or prospective data provider accesses the web server hosting MosquitoMap. A downloadable spreadsheet is available with instructions regarding data requirements, and data can be submitted via email. A link is also available to the map viewer hosted on an application server running ArcGIS Server 9.3 Enterprise SDE/SQL. The client can map and search the geodatabase, or use the Mal-area calculator (MAC) to quantify the overlap of models of vector, disease and human distribution. Output is available to the client in various formats.

The web server hosting the MosquitoMap home page provides access to historical accounts for particular datasets, information on how to contribute data, and metadata information for vector and pathogen models. The data portal within the MosquitoMap webpage leads to the application server containing the map viewer and mosquito collection database. Base maps featured in MosquitoMap include ESRI^® ^World satellite imagery and World Streetmap provided by ArcGIS Online Resource Centers [12]. MosquitoMap has pan and zoom controls, and is designed to operate similarly to other mass-market Internet mapping sites like Google Earth™. Mosquito collection point data are stored in an ArcGIS Server 9.3 Enterprise SDE/SQL Server 2005/2008 Standard geodatabase that includes GADM administrative area (see below) and vector species x country lookup tables, derived from the literature. Figure 1 shows the system architecture of MosquitoMap.

**Figure 2:**
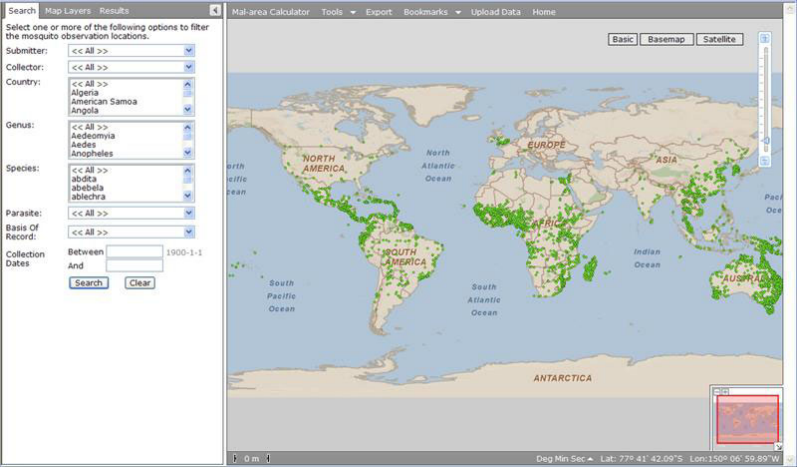
**Opening screen of the data viewer in the MosquitoMap web application showing the text based search area on the left and map viewer on the right**.

The opening screen of the data viewer has two panels, the left with a text 'search' function and links for 'map layer' and 'results', and the right with a map viewer and links, including for the 'Mal-area calculator' (Figure 2). The left panel allows a search against a selection of data fields (submitter, collector, country, genus, species, parasite, basis of record, and date). Following a search, a summary of the search results, including the number of observations, appears at the bottom of this panel. Simultaneously, a map of the collection locations from the search appears in the map panel. Clicking on the summary search results brings up the individual collection records (under 'results'). Information about the individual point data can be expanded, and the results exported as an MS Excel^® ^spreadsheet. A spatial search of point data can be done by selecting individual points on the map, or using the Tools>Identify feature to select an area containing mosquito collection records. In addition to mosquito collection points, results for GADM and populated places are also reported using a spatial search. Maps, including search results, can be exported as .kmz, .png, .jpg, .gif and .pdf formats, via the 'export' feature.

The 'map layer' link contains layers for mosquito point records, vector and disease models, and other layers of relevance to mosquitoes and vector borne disease (i.e. human population density, GADM administrative areas, populated places, streams, and water bodies - see below). These layers are designed to become active at different zoom levels, and their transparency is adjustable under 'Tools'. A preview feature is available under 'data uploader' to assist users to check the quality of their location data.

### Collection record input schema - point occurrences

MosquitoMap has over 60 fields of information [4], many of which are based on Darwin Core standards [13]. Data for some fields use controlled vocabulary terms based on international standards (e.g. ISO3166-1 for Country names). Information about a mosquito collection includes: submitter details; provenance, taxonomy, data and time details; geolocation details; specimen details; collection methodology; habitat details; and details about associated parasites, such as the detection of malaria sporozoites. One data field allows a record of the global unique identifier (GUID) associated with a collection record, i.e. a combination of institution code:collection code:catalog number. The GUID is designed to identify a record and can be reported separately in the literature.

MosquitoMap specimen records include vouchered material or observations only. Records can refer to individuals or to pools of individuals of the same species caught during the same collection event. Other information that does not have a dedicated field can be stored under the 'Remarks' field. The goal of the MosquitoMap application is to map specimen collections so, unlike a museum inventory, it is not primarily concerned about the derivatives of a single specimen, such as exuviae or genitalia, unless these are the sole representatives of the specimen. However, details about the derivatives of a single specimen can be noted under the 'Remarks' field. MosquitoMap reserves a category under CollectionCode for information about type specimens ('Type'), and the term 'LitRev' is used to flag information derived from the published literature. If a catalog number is unavailable, a temporary one is assigned, beginning with 'MMap'. The Institution code 'MMap' is also reserved for records, mainly from the literature, which cannot be readily prescribed to an institution.

A downloadable Excel spreadsheet is available via the 'Contribute Data' page of MosquitoMap that explains and lists the input data fields and controlled vocabulary terms for MosquitoMap, plus provides country and species lists for copying and pasting into new collection record databases. The spreadsheet provides advice to assist with data cleaning, and presently, the user must email their data to mosquitomap@si.edu. As mentioned above, a data preview tool is available to assist with maximizing data quality, e.g. to identify incorrect georeferences that do not fall on land or appear in the wrong country. The georeferencing and species identification accuracy of mosquito point occurrence data is important [4]. Every effort is made to ensure that the taxonomic data of the species within MosquitoMap is up-to-date using resources such as the Systematic Catalog of Culicidae [14], and the 'check coordinates' option in the program DIVA-GIS 5.3. The submitter name is offered as a searchable text data field within MosquitoMap partly to acknowledge the effort made to submit data. MosquitoMap is designed to share data within a framework of open access and due attribution, and data providers and users are asked to abide by the MosquitoMap data use agreement (see 'Contribute Data' page).

### Vector and pathogen/disease models

In addition to the layer containing mosquito collection points, other map layers are available including: human population density for 2000 [15]; Global Administrative Areas (version 0.9) [16], populated places [17], and water bodies and streams [17]. Disease models are available for Japanese encephalitis, dengue, Rift Valley fever, yellow fever [8], and malaria. The layers for malaria comprise spatial limits and endemic levels of *Plasmodium falciparum *in 2005 (hypoendemic, mesoendemic and hyper-holoendemic), and spatial limits and endemic levels of *P. vivax *in 2005 [18,19]. The malaria layers were digitized (0.09495°) from images that were available from the Malaria Atlas Project [20]. Errors of translation and geopositioning may have occurred, particularly in coastal areas, which were not present on the original image. As models of disease distribution become available to us, they will be hosted on MosquitoMap.

MosquitoMap currently includes distribution models for the Asian malaria vectors *Anopheles minimus *and *An. harrisoni *[6], and for the Korean species: *An. sinensis*, *An. kleini*, *An. belenrae*, *An. pullus*, *An. lesteri*, *An. sineroides*, *An. koreicus*, and *An. lindesayi *[7]. ESRI grid files for these vector distribution models are available for download via the MosquitoMap website. The vector maps for Korea represent the entire anopheline mosquito fauna for that country and thus provide the most complete information for testing the MAC concept (see below).

### Mal-area Calculator

**Figure 3:**
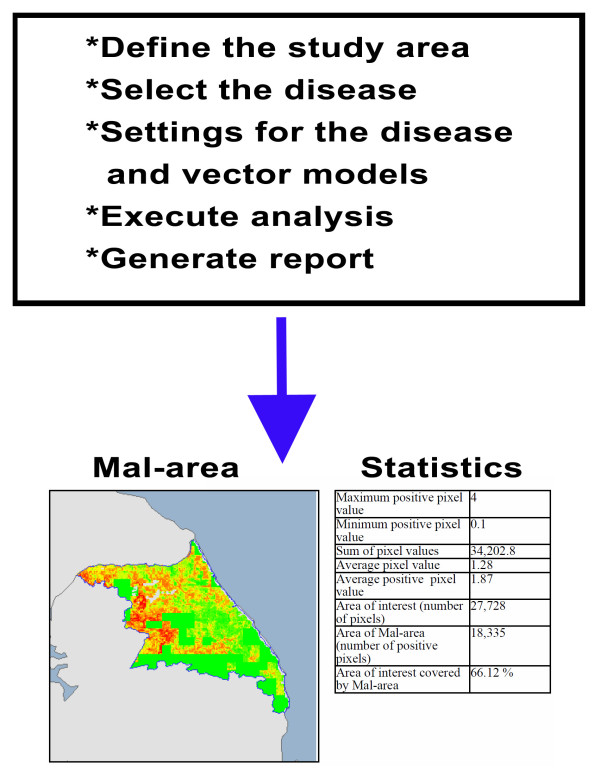
**Representation of the steps involved in the Mal-area calculation within the web application MosquitoMap, for an area of interest in South Korea**.

MosquitoMap includes the MAC application, which is a raster overlay analytic tool for comparing human, mosquito, and pathogen/disease distribution layers. ArcGIS^® ^ModelBuilder™ 9.3[21] was chosen for the MAC computations. The design process in ArcGIS ModelBuilder is essentially the same as creating a flow diagram that mimics the actual tasks that would be performed manually. A menu driven interface for the MAC was designed and developed in the programming language C# .NET for the ArcGIS Server™ 9.3 Web ADF. The user interface was designed in 5 consecutive pop-up menus, followed by a prompt for the user to start the calculation. Once started, menus progress, as in Figure 3.

The first pop-up menu allows the user to define the AOI for the pathogen and vector models. When a country is chosen this filters a global table of mosquito vector species, so that only pathogen and distribution models for mosquito species listed for the country are considered in the calculations. The user can further refine the AOI by choosing one of the GADM second order administrative areas, or use the area selection toolbar to apply a polygon selection. To improve performance, vector and disease rasters that overlap the AOI were first converted to vector format using ArcToolbox™ and then merged into a set of contiguous polygons for each disease. For each polygon, an attribute was created with a URL linking to the original raster dataset, provided for the Mal-area calculation.

**Figure 4:**
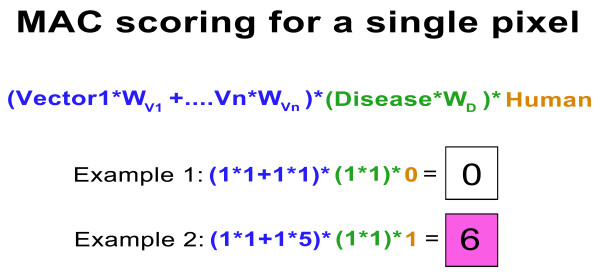
**Map algebra expression used in the Mal-area calculator (MAC) within the web application MosquitoMap**. Input values for different vectors (V1, V2 etc), disease (D) and Human (H) is 0 or 1, and weightings for vectors (W_V1 _to W_Vn_) or disease (W_D_) are integer values of 1 to 10. Note if humans, vector or disease is absent for a pixel the result for that pixel is zero (example 1). Example 2 shows how the pixel value increases if one of the vectors is given a higher weight due to its vectorial importance. Input values for vector, disease and Human is 0 or 1, and weights are integer values of 1 to 10.

In the second pop-up menu, the user selects the vector-borne disease of interest, which leads to the third pop-up menu, where the user selects from the available pathogen and vector distribution models. The user can also adjust the weights assigned to pathogen and vector distribution models via a slide bar that applies integer values between 1 and 10. This facility allows user-defined weightings to be incorporated into the calculations, such as when primary and secondary vectors occur in the same AOI. Vectorial importance can vary depending on a variety of factors including the blood-feeding habit, longevity, and abundance [22]. The weightings represent a composite of these factors, and are a relative rather than absolute measure. It should be noted that a mosquito species may be a primary vector in one AOI but not another, and the user can adjust the weighting accordingly. Also, different weights can be applied to the pathogen distribution model, or elements of the pathogen model, e.g. hypo-, meso- and hyper-endemic extents of models for *P. falciparum*. The map algebra expression used in the MAC is shown in Figure 4.

Vector and pathogen distribution maps are usually classified according to probability of occurrence (integers of 1-100), number of models supporting occurrence (typically 1-10), or as absence and presence (0, 1). These different categorizations can lead to normalization issues and complicate the meaning of the output. To improve computation efficiency and simplify the output of the MAC, a threshold is applied to all input layers to render them as presence or absence. For example, the least presence threshold [23] may be applied to vector distribution models, and a 50% probability threshold for disease models.

To simplify calculations, the human population density layer was pre-defined as a binary (presence, absence) feature class, and the presence threshold was set to one or more people per square kilometer. This threshold has been used to mask low human population densities in previous calculations of malaria distribution [18,19]. As map layers occur in different resolutions, all layers were resampled to the resolution of the human population density layer (0.008333°). The value of the resampled pixel was determined by the value at its center. 'No data' values were not included in the Mal-area calculation for the AOI.

**Figure 5:**
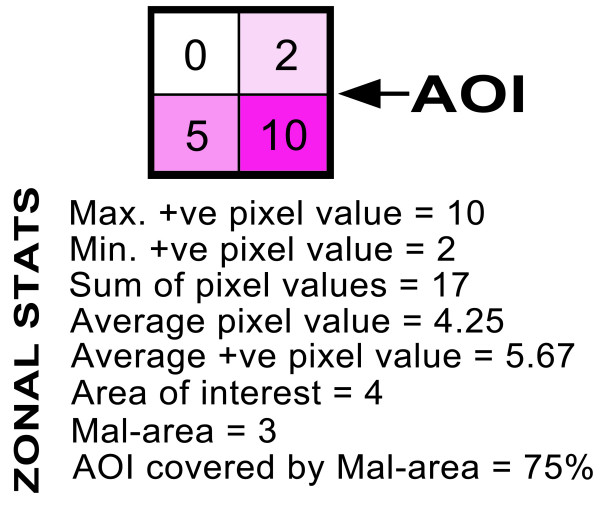
**Example of zonal statistics output for an area of interest (AOI) from the Mal-area calculator (MAC) within the web application MosquitoMap**. Note the coverage of the AOI that is the Mal-area (colored area), i.e. the percentage of the AOI where disease transmission is theoretically possible. This statistic can provide a relative index of risk when compared to another AOI.

The output of the MAC is a .pdf containing a list of the input parameters, and map outputs for the AOI of the Mal-area, the pathogen model, the vector models, and a human population density map. Each map is accompanied by statistics derived from a table of zonal statistics from the ModelBuilder for the AOI (Figure 5). The Mal-area output statistics are: 1) maximum positive pixel value; 2) minimum positive pixel value; 3) sum of pixel values; 4) average pixel value (for all pixels); 5) average positive pixel value (i.e. ignoring zero value pixels); 6) area of interest (number of pixels); 7) Mal-area (number of positive pixels); 8) area of interest covered by the Mal-area (as a percentage). For disease, vector and human density outputs, output statistics refer to the areas covered by these respective layers.

A calculation can be made for the same AOI with different input parameters, or for another AOI, to compare the resulting Mal-area estimate. In as much as the Mal-area can be seen as an estimate of disease transmission risk, this process quantifies the relative risk of different AOIs.

## Results and Discussion

Mosquito occurrence is tied to the environment. For example, all mosquitoes have an aquatic larval stage, do not occur above a certain elevation, and need a minimum amount of solar radiation to develop. It is tempting to regard vector species as a homogeneous component of the environmental background to disease transmission. However, mosquito species can utilize different microclimates, vary in their biting behavior, and may have diverse life histories. An additional complication is that a species may be a primary vector in one area of its range but, for reasons that are unclear, may play a secondary role in other areas. It has been proposed that a more objective understanding of regional differences in the underlying force of malaria transmission can be attained by considering the properties intrinsic to the regionally most "dominant" vector species [22]. We suggest that any attempt to understand the spatial distribution of vector-borne disease transmission will benefit from knowledge of the distribution and vectorial importance of the entire vector species fauna within an AOI. Thus, a goal of MosquitoMap is to provide awareness, not just of the potential extents of individual mosquito species, but of the implications for disease transmission of the combination of vector spatial distributions within an AOI.

The Mal-area concept emphasizes the independent but cumulative role of vector species in determining disease risk. Many other factors affect disease transmission but are not considered in the MAC, for example, health care coverage, host immunity, degree of vector control or insecticide treated bednet usage, and insecticide and drug resistance. It is anticipated that mosquito and pathogen distribution models will vary in their accuracy and biological realism, which will affect estimates of disease risk. For example, vector ecological niche or habitat suitability models are often based on presence only data, and are affected by the number of data points, selection of environmental layers, and the algorithms used to generate the predictions. Although the prediction of vector species spatio-temporal dynamics is possible [24], ecological niche models are usually constructed to predict static average yearly spatial extents, and potential presence rather than abundance. However, as models are improved and are made available on MosquitoMap, the biological realism of the MAC output should also improve. The MAC allows users, such as vector disease control workers, preventative medicine practitioners, and health planners, to see within the AOI where the predicted transmission risk areas are located, and quantifies this risk area for comparison with other AOI. In the absence of other intelligence information, the MAC can provide a useful initial decision tool, affecting such things as: counselling for prophylaxis, choice of health messages, where best to locate personnel, the form of vector control, and the vector identification tools needed.

**Figure 6:**
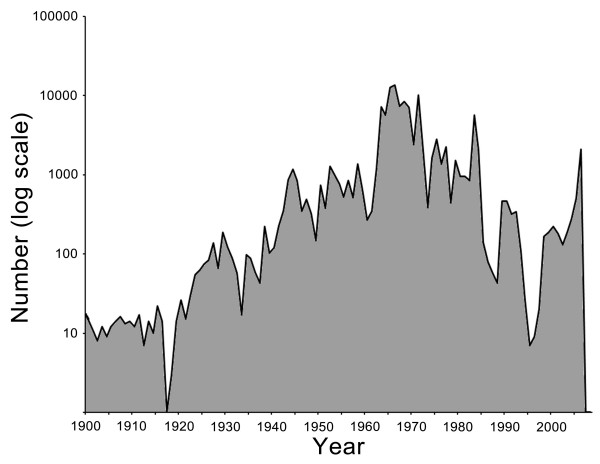
**Frequency distribution of the dates of mosquito collection records within the web application MosquitoMap as on Oct 2009**.

MosquitoMap [11] is publicly available and, as of Oct, 2009, contained 126,031 mosquito collection records. These records pertain to 1,891 scientific names (complexes, subspecies, species and above) from 143 countries (see Figure 6). The oldest record, for the type locality of *Toxorhynchites haemorrhoidalis*, is from 1787. Most records date from the mid 1960s to early 1970s, due mainly to records from the Mosquito Information Management Project [2,3].

Point data in MosquitoMap could have a variety of uses including: informing medical entomologists about where mosquito collection efforts should be directed; identifying areas relevant to the study of mosquito biogeography, evolution and biodiversity; allowing predictions about the potential spread of exotic mosquito introductions; allowing predictions about the potential effects of global warming on mosquito distributions; allowing insights into mosquito community structure, and environmental and climatic correlates to species occurrence (ecological niche); allowing continent-wide rather than just local studies of vector-borne disease; and identifying cryptic evolutionary lineages that differ in geographic or ecological space.

MosquitoMap could be expanded to include other environmental layers relevant to mosquito distribution (e.g. coastal forest urban, forest fringe, rice irrigation) or vector-borne diseases (urban/rural, impregnated bed nets, war zone, drug resistance, border areas, immunological status, distance to health clinic). Some challenges common to disease mapping [25] include: the heterogeneity of data sources, and difficulties in integrating data from MosquitoMap with other disease management and biodiversity systems. We hope to improve the functionality of MosquitoMap, and to use it as a template for other vectors (e.g. sand flies, ticks and fleas) of disease.

## Conclusion

We developed a Web-based spatial database of mosquito collection records and distribution models called MosquitoMap. An application within MosquitoMap, called the MAC, quantifies the area of overlap, for any AOI, of vectors, humans and disease. MosquitoMap and the MAC can be utilized by medical entomologists, vector disease control workers, preventative medicine practitioners, and health planners to determine what species have been collected where, and to estimate the Mal-area for vector-borne diseases risk assessment. As more users submit records and distribution maps, the utility of these online resources will increase. Data on MosquitoMap are freely available and contributions are clearly sourced and acknowledged within MosquitoMap using appropriate citations provided by the contributor.

## Competing interests

The authors declare that they have no competing interests.

## Authors' contributions

MosquitoMap was made possible from a Global Emerging Infections Surveillance and Response System (a Division of the Armed Forces Health Surveillance Center - AFHSC/Div of GEIS Ops) grant obtained by LR (Principal Investigator), RW and DF. DF and RW conceived the idea of MosquitoMap and the MAC, JC, IB and SH constructed the applications in consultation with DF and RW. DF drafted the manuscript. All authors read and approved of the final manuscript.
